# A Novel Transcription Factor *CaSBP12* Gene Negatively Regulates the Defense Response against *Phytophthora capsici* in Pepper (*Capsicum annuum* L.)

**DOI:** 10.3390/ijms20010048

**Published:** 2018-12-22

**Authors:** Huai-Xia Zhang, Muhammad Ali, Xiao-Hui Feng, Jing-Hao Jin, Liu-Jun Huang, Abid Khan, Jing-Gang Lv, Su-Yan Gao, De-Xu Luo, Zhen-Hui Gong

**Affiliations:** 1 College of Horticulture, Northwest A&F University, Yangling 712100, China; 2016060124@nwsuaf.edu.cn (H.-X.Z.); alinhorti@yahoo.com (M.A.); fengjn1555@163.com (X.-H.F.); Jinjinghao123@126.com (J.-H.J.); 18792575048@126.com (L.-J.H.); abidagriculturist@gmail.com (A.K.); 2 Tianjin Vegetable Research Center, Tianjin 300192, China; lvjinggang1963@163.com (J.-G.L.); 13920170536@163.com (S.-Y.G.); 3 Xuhuai Region Huaiyin Institute of Agricultural Sciences, Jiangsu 223001, China; luodexu2002@163.com

**Keywords:** pepper, *CaSBP12*, *Phytophthora capsici*, defense-related genes, *Nicotiana benthamiana*

## Abstract

SBP-box (Squamosa-promoter binding protein) genes are a type of plant-specific transcription factor and play important roles in plant growth, signal transduction and stress response. However, little is known about the SBP-box genes in pepper (*CaSBP*), especially in the process of *Phytophthora capsici* infection. In this study, a novel gene (*CaSBP12*) was selected from the *CaSBP* gene family, which was isolated from the pepper genome database in our previous study. The *CaSBP12* gene was located in the nucleus of the cell and its silencing in the pepper plant enhanced the defense response against *Phytophthora capsici* infection. After inoculation with *Phytophthora capsici*, the root activity of the *CaSBP12*-silenced plants is compared to control plants, while malondialdehyde (MDA) content is compared viceversa. Additionally, the expression of defense related genes (*CaPO1*, *CaSAR8.2*, *CaBPR1,* and *CaDEF1*) in the silenced plants were induced to different degrees and the peak of *CaSAR8.2* and *CaBPR1* were higher than that of *CaDEF1.* The *CaSBP12* over-expressed *Nicotiana benthamiana* plants were more susceptible to *Phytophthora capsici* infection with higher EC (electrical conductivity) and MDA contents as compared to the wild-type. The relative expression of defense related genes (*NbDEF*, *NbNPR1*, *NbPR1a,* and *NbPR1b*) in transgenic and wild-type *Nicotiana benthamiana* plants were induced, especially the *NbPR1a* and *NbPR1b*. In conclusion, these results indicate that *CaSBP12* gene negative regulates the defense response against *Phytophthora capsici* infection which suggests their potentially significant role in plant defense. To our knowledge, this is the first report on *CaSBP* gene which negative regulate defense response.

## 1. Introduction

Pepper (*Capsicum annuum* L.), widely cultivated throughout the world, is an important vegetable crop with high economic value, but it is prone to damage by diseases and insects, especially *Phytophthora* blight caused by a pathogen *Phytophthora capsici* (*P. capsici*) [1]. *Phytophthora capsici* is a soil-borne disease. It can infect roots, stems, leaves, fruits of pepper plant and also other crop plants, such as tomato, eggplant, cucumber, watermelon, pumpkin, snap peas, and lima beans [2,3]. To combat pathogenic attack, plants have developed sophisticated defense mechanisms during co-evolution with pathogen infection [4]. One of the mechanisms is inducible innate immunity, which is largely regulated at the transcriptional level by the actions of many transcriptional factors (TFs). TFs play an important role in the regulation of gene expression networks. It has been reported that TFs are promising biotechnological targets for engineering plants with improved disease resistance [5,6,7]. SBP-box (Squamosa-promoter binding protein) genes are specific to plants and encode a class of zinc finger-containing transcription factors with a broad range of functions. The SBP-box genes named *SBP1* and *SBP2* were identified for the first time in *Antirrhinum majus* [8]. The SBP-box genes were later identified in numerous other plants such as *BpSPL1* in *Betulapendula* [9], *AtSPL14* in *Arabidopsis* [10], and *VpSBP5* in *Vitis vinifera* [11]. Previously, it was believed that SBP-box genes are related to plant growth and development, but recent studies showed that SBP-box genes play an important role in signal transduction as well as abiotic and biotic stress. For instance, *AtSPL14,* involved in programmed cell death was induced by the fungi producing fumonisin B1 [10]. *AtSPL2* (At5g43270), which is modified in transgenic *Arabidopsis* over-expresses the *JASMONATE CARBOXYL METHYLTRANSFERASE* gene *(AtJMT)* in response to the jasmonic acid mediated resistance pathway [12]. *VpSBP5* likely participates in regulating resistance to *Erysiphe necator* by activating the SA-induced systemic acquired resistance pathway and MeJA-induced wound signaling pathway in grapes [11]. It has been reported that the SBP-genes of *Brassica rapa* and *Chrysanthemum* also respond to hormonal treatments [13,14]. As the stable transformation in pepper plants remains a challenge so the method of virus-induced gene silencing (VIGS) is widely used in pepper plants to identify the gene’s functions. Previously Abid et al., and Zhang et al. identified the genes functions of *CaDIR7* and *CaRGA2* by using VIGS techniques after the infection of *P. capsici* [15,16]. In addition, transgenic technology is also widely using to study and identified the functions of gene’s function, for example overexpression of *CaTIP1-1* gene in tobacco enhances resistance to osmotic stresses [17]. However, the function of the SBP-box family genes in pepper against the infection of *P. capsici* is unknown to date.

*CaSBP12* gene (Accession no. Capana10g000886) with an open reading frame of 900bp, encoding 299 amino acids is a SBP-box gene in the pepper plant. It contains a highly conserved DNA-binding domain termed the SBP domain. This domain comprises approximately 76 amino acid residues that are involved in both DNA binding and nuclear localization including two zinc-binding sites [18]. In our previous research work, we found that the expression of *CaSBP12* can be significantly and differentially modulated during compatible and incompatible interactions with *P. capsici* [18]. Additionally, the expression of *CaSBP12* can be inhibited by the salicylic acid (SA) and methyl jasmonate (MeJA) synthesis inhibitor and induced by SA and MeJA [18]. Therefore, we selected this gene to further elucidate its function against *P. capsici* infection and also its localization in the cell.

## 2. Results

### 2.1. *CaSBP12* Protein is Localized in the Nucleus

**Figure 1:**
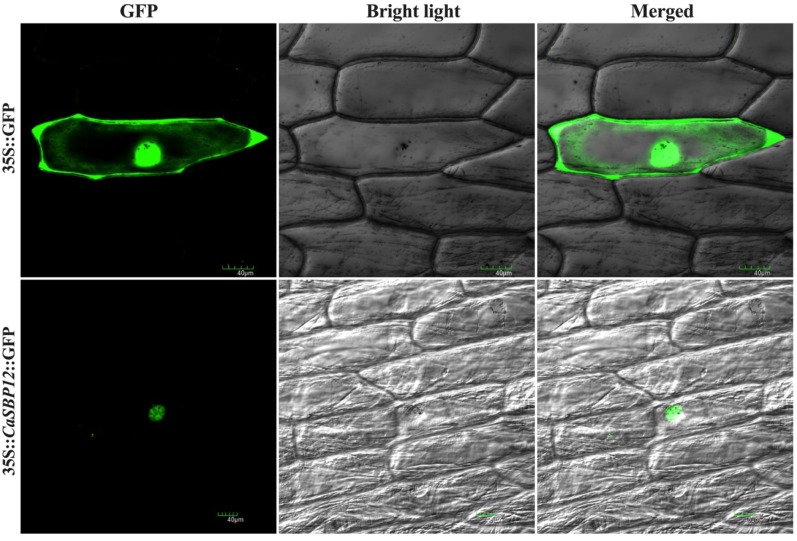
Subcellular localization of the CaSBP12 protein. Transient expression of 35S::GFP, 35S::*CaSBP12*::GFP in onion epidermal cells via particle bombardment. The fluorescence was visualized using a laser scanning confocal microscope under bright and fluorescence fields. The photographs were taken in a dark field for green fluorescence and under bright light for the morphology of the cell. Bars in this picture are 40μm.

To determine the subcellular localization of CaSBP12 protein, the 35S::*CaSBP12:*:GFP and 35S::GFP (used as a control) plasmids were transiently expressed in onion epidermal cells. The results indicated that the control 35S::GFP exhibited GFP signals in the whole cell, including the nucleus, cytoplasm, cell membrane, and cell wall, while 35S::*CaSBP12:*:GFP only exhibited GFP signals in the nucleus (Figure 1). This indicated that *CaSBP12* was located in the nucleus of the cell.

### 2.2. Silencing the CaSBP12 Gene Enhanced Pepper Plant Resistance to P. capsici Infection

#### 2.2.1. Phenotypic Observation and Silencing Efficiency of *CaSBP12* Gene Silencing Plants

**Figure 2:**
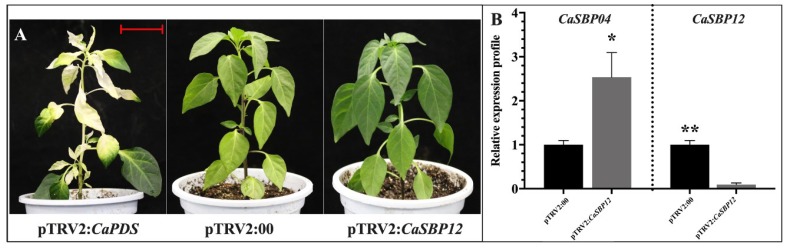
Phenotypes and silencing efficiency of *CaSBP12* in silenced and control plants. (**A**) Phenotypes of *CaPDS*-silenced, *CaSBP12*-silenced, and control plants. (**B**) Silencing efficiency of *CaSBP12* and comparative expression of *CaSBP04* (a highly homologous gene of *CaSBP12*) in the silenced and control plants. The red line used as a scale bar (length 3 cm). The means were analyzed using the least significant difference (LSD). * represents significant difference at *p* ≤ 0.05, and ** represents highly significant difference at *p* ≤ 0.01. Mean values and standard errors (SEs) for three replicates are shown.

In this study, a positive control vector (pTRV2:*CaPDS*) was used for the silencing of the *CaPDS* gene (GenBank accession number: X68058), which produced a typical white color as a result of the photo-bleaching phenotype in the leaves, while the negative control was pTRV2:00. The method of leaf injection and root drench was used for the silencing of *CaSBP12*. Forty-five days post inoculation, the positive control (pTRV2:*CaPDS*) plants showed photo-bleaching, while the pTRV2:00 and *CaSBP12*-silenced plants exhibited no phenotypic changes (Figure 2A); we then detected the silencing efficiency of *CaSBP12.* In order to ensure the silencing specificity of *CaSBP12*, the relative expression of *CaSBP04* (SGN locus: Capana01g003445, a member of pepper SBP gene family, having the highest homology with *CaSBP12*), which is related to it in the negative control plants, was also measured in the *CaSBP12-*silenced plants. As shown in Figure 2B, the silencing efficiency of *CaSBP12* was up to 90%, while the expression of *CaSBP04* in the pTRV2:*CaSBP12* plants were up-regulated as compared with pTRV2:00 plants. This indicated that *CaSBP12* was successfully, specifically silenced.

#### 2.2.2. Identification of *CaSBP12* Gene Involvement in *P. capsici* Resistance 

**Figure 3:**
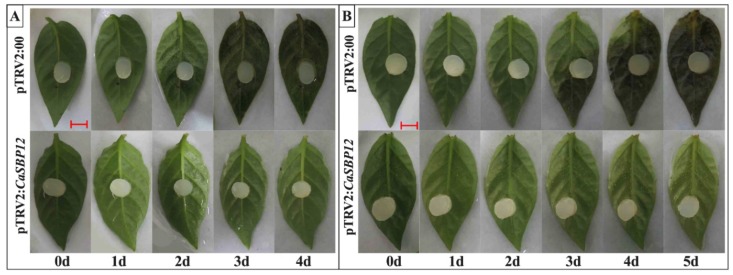
Phenotypes of detached leaves of the *CaSBP12*-silenced and control plants after inoculation with *P. capsici* HX-9 (**A**) or PC (**B**) strains. (d: represents day). The red line used as a scale bar (length 0.8 cm).

After detected the silencing efficiency of *CaSBP12*, the detached leaves of the silenced and control plants were inoculated with the compatible (HX-9) and incompatible (PC) strains of *P. capsici.* After inoculation with the HX-9 strain, the detached leaves of the control plants exhibited small hygrophanous lesion areas at the second day, water-soaking at the third day, and the edge of the leaf blade showed chlorosis and the initial signs of decay at 4 dpi (days post inoculation). The detached leaves of *CaSBP12*-silenced plants showed no disease symptom during this period (Figure 3A). After inoculation with the PC strain, the detached leaves of the control plants showed water-soaking at 3rd day, which expanded to four days, and the whole leaves appeared to have diseased symptoms and the initial signs of decay at day five. The detached leaves of *CaSBP12*-silenced plants showed no disease symptoms during this period (Figure 3B). It is worth noting that the disease developing speed of the detached leaves inoculated with HX-9 strain is faster than that inoculated with the PC strain. This indicated that *CaSBP12* may negatively regulate the defense response against *P. capsici* in pepper.

**Figure 4:**
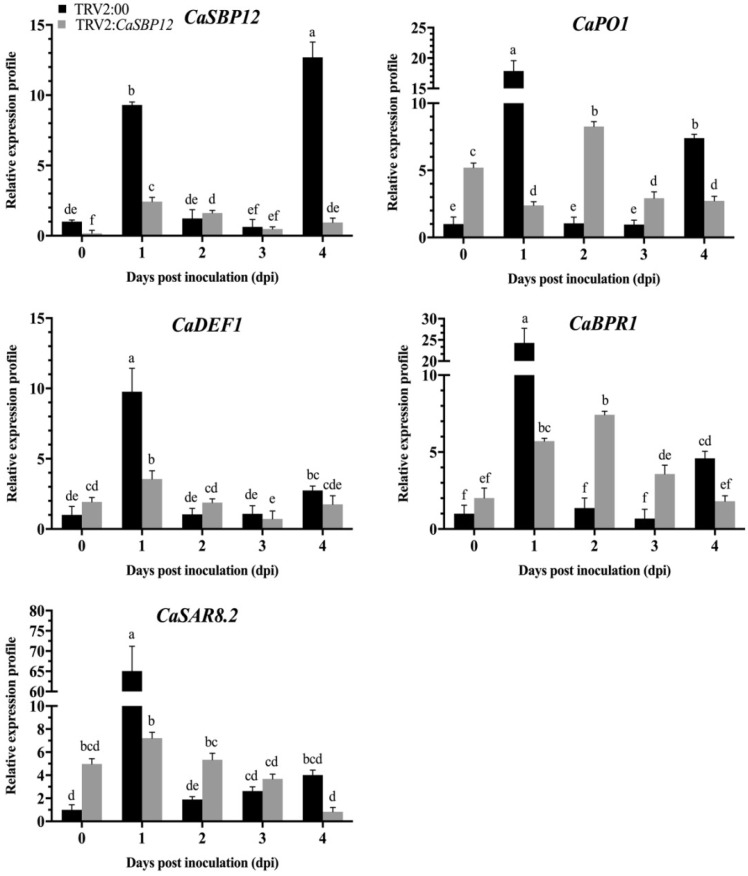
The expression of *CaSBP12* and defense related genes after inoculation with the HX-9 strain of *P. capsici* in silenced and control plants. After measuring the silencing efficiency of the *CaSBP12* gene, 5 mL of 1 × 10^5^ cfu/mL zoospore of *P. capsici* were used to inoculate the silenced and control plants, respectively, using the root-drench method, and then roots of silenced and control plants were collected at 0, 1, 2, 3, and 4 d for the detection of defense related genes. *CaPO1 (*Accession number: AF442386); *CaDEF1* (Accession number: AF442388); *CaBPR1* (Accession number: AF053343); *CaSAR8.2* (Accession number: AF112868). The means were analyzed using the least significant difference (LSD). Bars with different lower-case letters indicate significant differences at *p* ≤ 0.05. Mean values and SEs for three replicates are shown.

*P. capsici* is a soil-borne disease and is known to infect roots, stems, leaves, flowers and fruits. Moreover, the disease progression is easier to observe in roots. Additionally, the expression level of *CaSBP12* is higher in the roots [18]. Therefore, we measured the expression level of some defense related genes i.e. *CaPO1* (peroxidase), *CaSAR8.2* (systemic acquired resistance), *CaBPR1* (pathogenesis-related (PR)-1 protein) and *CaDEF1* (defensin) in roots. The expression level of *CaSBP12* and defense-related genes (*CaPO1*, *CaSAR8.2*, *CaBPR1,* and *CaDEF1*) in the roots of the silenced and control plants were measured after inoculation with HX-9 and PC strains of *P. capsici*. As shown in Figure 4, the expression of *CaSBP12* in the control and *CaSBP12*-silenced plants increased at first and then decreased after inoculation with the HX-9 strain, but the expression level of *CaSBP12* in the silenced plants (except day two and day three) was obviously lower than that in control plants. The defense related genes (*CaPO1*, *CaSAR8.2*, *CaBPR1*, and *CaDEF1*) exhibited the same expression patterns in the control plants with peak expression levels reached at day one, and then the expression decreased and increased again at day four. At day zero, day two, and day three, the expression of the defense-related genes (except *CaDEF1*) in the *CaSBP12*-silenced plants is higher than the control plants. It is notable that the defense-related genes in the control plants peaked early or at the same time as *CaSBP12*-silenced plants, but the expression levels were significantly higher than that in the silenced plants. 

**Figure 5:**
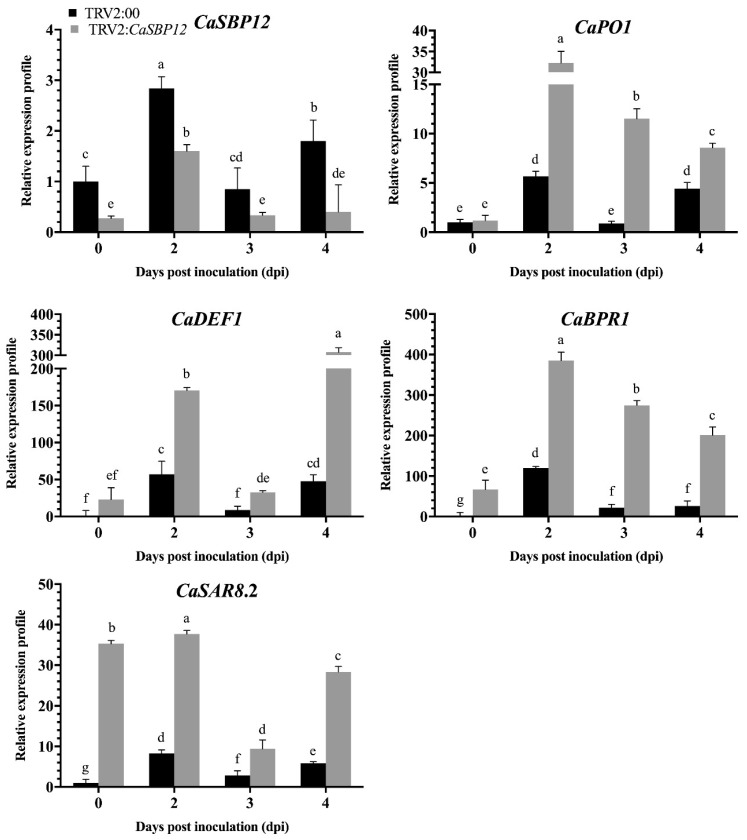
The expression of *CaSBP12* and defense related genes after inoculation with the PC strain of *P. capsici* in silenced and control plants. After measuring the silencing efficiency of the *CaSBP12* gene, 5 mL of 1 × 10^5^ cfu/mL zoospore of *P. capsici* were used to inoculate the silenced and control plants, respectively, using the root-drench method, and then roots of silenced and control plants were collected at 0, 2, 3, and 4 d for the detection of defense related genes. The means were analyzed using the least significant difference (LSD). Bars with different lower-case letters indicate significant difference at *p* ≤ 0.05. Mean values and SEs for three replicates are shown.

PC strain post-inoculation, the expression level of *CaSBP12* in control plants increased and was significantly higher than in the silenced plants (Figure 5). The expression of the defense-related genes (*CaPO1*, *CaSAR8.2*, *CaBPR1*, and *CaDEF1*) was increased in control and silenced plants, while the expression level peaked at 2 dpi except *CaDEF1* which showed higher expression at 4 dpi (Figure 5). The expression of *CaPO1*, *CaSAR8.2*, *CaBPR1,* and *CaDEF1* genes in the silenced plants were significantly higher than in the control plants. Collectively, the expression of the defense-related genes in the silenced plants inoculated with the PC strain was higher than that inoculated with HX-9 strain. It is notable that the expression of *CaPO1, CaSAR8.2, CaBPR1,* and *CaDEF1* in the silenced plants was induced to different degrees, especially the expression of *CaSAR8.2* (inoculated with the HX-9 strain), and *CaBPR1* (inoculated with the PC strain). This indicated that *CaSBP12* may be involved in plant resistant to *P. capsici* in pepper.

#### 2.2.3. Determination of Root Activity of Gene-Silencing Plants

**Figure 6:**
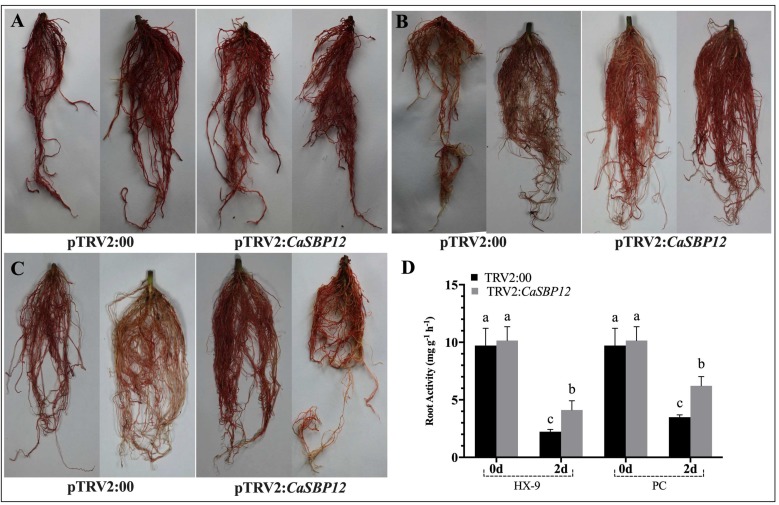
Determination of root activity of the silenced and control plants after inoculation with *P. capsici* strains (**A**) Phenotypes of the silenced and control plant roots stained with triphenyl-tetrazolium chloride (TTC) after inoculation with *P. capsici* at 0 d. (**B**) Phenotypes of the silenced and control plant roots stained with TTC after inoculation with the HX-9 strain of *P. capsici* at 2 d. (**C**) Phenotypes of the silenced and control plant roots stained with TTC after inoculation with the PC strain of *P. capsici* at 2 d. (**D**) Roots activity of the silenced and control plants after inoculation with *P. capsici* strains at 2 d. The means were analyzed using the least significant difference (LSD). Bars with different lower-case letters indicate significant differences at *p* ≤ 0.05. Mean values and SEs for three replicates are shown.

Triphenyl-tetrazolium chloride (TTC) staining is the reaction of TTC with succinate dehydrogenase in mitochondria of living cells, which produces red methylene, which is used to express cell viability. Therefore, the TTC staining method was used to determine the root activity of gene silenced plants that were inoculated with the HX-9 and PC strains. After being stained with the TTC, no obvious difference was recorded in the root color of *CaSBP12*-silenced and control plants at day zero (Figure 6A). However, the color of the *CaSBP12-*silenced plant roots are darker than the control plant roots at the second day (Figure 6B,C). Moreover, the root activity of *CaSBP12*-silenced plants showed a higher root activity as compared to control at day two (Figure 6D). These results are consistent with the aforementioned from in vitro leaves showing resistance, i.e., the silencing of *CaSBP12* enhance the resistance of pepper plants against *P. capsici*. It is notable that after inoculation with the HX-9 strain, the root activity of the gene-silenced and control plants decreased more than that of the plants inoculated with the PC strain (Figure 6D).

#### 2.2.4. Determination of Malondialdehyde (MDA) Content

**Figure 7:**
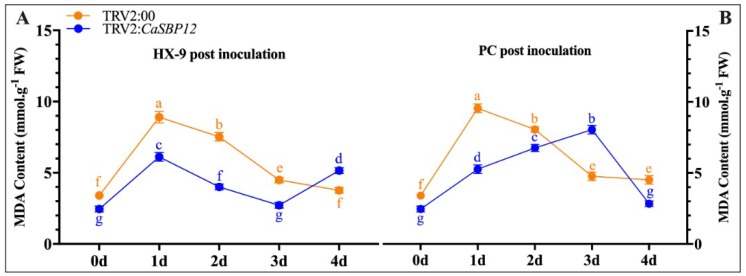
The malondialdehyde (MDA) content of the silenced and control plants after inoculation with *P. capsici* strains. (**A**) MDA content after inoculation with the HX-9 strain of *P. capsici* in silenced and control plants. (**B**) MDA content after inoculation with the PC strain of *P. capsici* in silenced and control plants. Bars with different lower-case letters indicate significant differences using the least significant difference (LSD) value (*p* ≤ 0.05). Mean values and SEs for three replicates are shown.

A plant under stress is closely related to membrane lipid peroxidation, which is induced by active oxygen accumulation. MDA is one of the most important products of membrane lipid peroxidation. Therefore, the degree of membrane lipid peroxidation can be determined by measuring the content of MDA. This can reflect the degree of damage of the membrane system and the resistance of the plant [19]. Therefore, the MDA content was measured in both *CaSBP12-*silenced and control plants after inoculation with the HX-9 and PC strains of *P. capsici.* The MDA content was increased in *CaSBP12-*silenced plant after inoculation with the HX-9 and PC strains (Figure 7). After inoculation of both strains HX-9 and PC, the content of MDA was higher in control plants as compared to *CaSBP12*-silenced plant except 4-dpi in HX-9 (Figure 7A) and 3-dpi in PC stain (Figure 7B). Changes in the MDA content of the control plants showed same trends after inoculation of both HX-9 and PC strains. Compared to the HX-9 strain, the peak time after inoculation with the PC strain appeared later in the *CaSBP12*-silenced plants. These results indicated that the *CaSBP12*-silenced plants were less damaged than the control after inoculated with *P. capsici* and the damage reduction after the silencing of *CaSBP12*, was higher in plants inoculated with the PC strain compared to those inoculated with the HX-9 strain.

#### 2.2.5. Disease Index Percent Statistics

**Figure 8:**
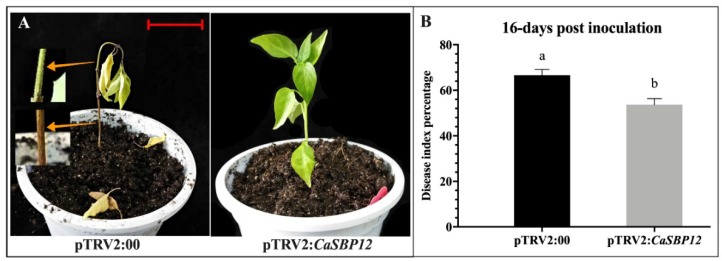
Phenotypes and disease index percent of the silenced and control plants after inoculation with the HX-9 strain of *P. capsici.* (**A**) Phenotypes of the silenced and control plants after inoculation with the HX-9 strain of *P. capsici*. The two yellow arrows indicated the constricted area of the stem. (**B**) Disease index percent of the silenced and control plants after being inoculated with the HX-9 strain of *P. capsici*. Photographs and disease index percent were counted and taken at 16-day post-inoculation, respectively. The red line is used as a scale bar (length 3 cm). Bars with different lower-case letters indicate significant differences using the least significant difference (LSD) value (*p* ≤ 0.05). Mean values and SEs are shown.

As Figure 3, Figure 6, and Figure 7 showed, *CaSBP12* plays a negative regulatory role in the process of *P. capsici* infection. Therefore, the disease index percent was also calculated after inoculation with the HX-9 strain using the root-drench method as described by Wang (2013) [20]. After inoculation with the HX-9 strain, the infection was monitored at day 16. As shown in Figure 8A, the silencing of *CaSBP12* enhanced disease resistance against *P. capsici* compared to non-silenced plants. The disease of *CaSBP12*-silenced and control plants was quantified. As shown in Figure 8B, the disease index percent of the *CaSBP12*-silenced plants was significantly lower than the control plants. These results indicated that the silencing of *CaSBP12* enhanced the resistance against *P. capsici* infection.

### 2.3. Overexpression of CaSBP12 in Nicotiana Benthamiana Enhanced Susceptibility to P. capsici Infection

#### 2.3.1. Disease Resistance Identification of *CaSBP12* Overexpression in Plants

**Figure 9:**
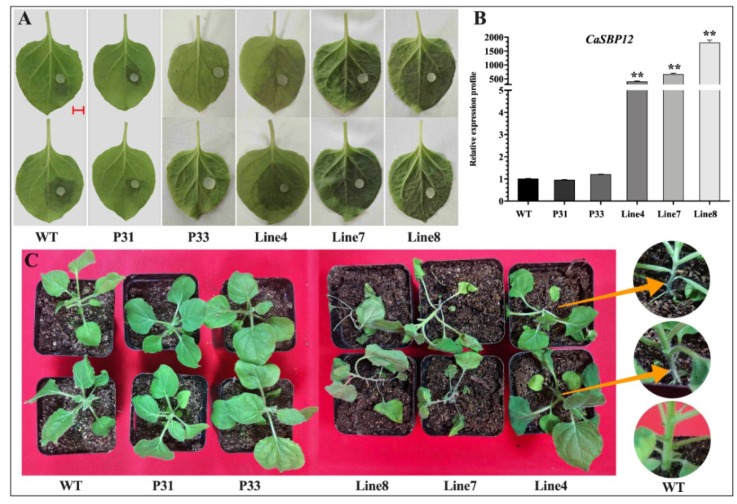
Phenotypes of the wild-type (WT), empty vector (P31, P33) and transgenic lines (line 4, line 7, and line 8) after inoculation with *P. capsici* and the expression of *CaSBP12* in the transgenic, empty vector, and wild-type lines of *N. benthamiana*. (**A**) Phenotypes of the detached leaves of transgenic, empty vector, and wild-type lines after inoculation with *P. capsici.* (**B**) The expression of *CaSBP12* in the transgenic, empty vector, and wild-type lines of *N. benthamiana.* The expression levels were analyzed between the transgenic lines (line 4, line 7, and line 8) and the wild-type (WT). (**C**) Phenotypes of the transgenic, empty vector, and wild-type (WT) lines after inoculation with *P. capsici*. The yellow arrows indicate the constricted area of the stem. Forty-five-day-old *N. benthamiana* plants were used for this experiment. The red line used as a scale bar (length 0.4 cm). The diameter of the plug of *P. capsici* used in this experiment is 0.4 cm. The diameter of the pot in Figure 9C is 7 cm. The means were analyzed using the least significant difference (LSD). Double asterisks (**) represents highly significant difference at *p* ≤ 0.01. Mean values and SEs for three replicates are shown.

In order to further confirm the role of *CaSBP12* in the process of *P. capsici* infection, we generated transformed *Nicotiana benthamiana (N. benthamiana*) plants overexpressing *CaSBP12* using *A. tumefaciens*-mediated leaf disc transformation because the stable transformation of pepper plants remains challenging. Eight numbers of transgenic lines were obtained, and there was no difference in phenotypes as compared with the wild-type plants. Two transgenic lines with empty vectors (P31 and P33) and three transgenic lines over-expressing *CaSBP12* gene (line 4, line 7, and line 8) were randomly selected for disease resistance identification. Forty-day-old seedlings were used for this experiment. After inoculation with the HX-9 strain of *P. capsici*, the detached leaves of wild-type plants and empty vector transgenic lines (P31 and P33) showed a small or no hygrophanous lesion area at 2nd day, while the hygrophanous lesion area occupied almost two-thirds of the leaves from the transgenic lines (line 4, line 7, and line 8) (Figure 9A). The diameter of the leaf lesions of the transgenic lines (line 4, line 7, and line 8) was higher than that of wild-type plants (Supplementary Figure S1). However, the leaf lesions diameter of transgenic lines (line 4, line 7, and line 8) was not significantly different from each other which can be seen in supplementary Figure S1. The expression of *CaSBP12* in the transgenic lines (line 4, line 7, and line 8) was different as compare to wild-type (WT) and empty vector (P31, P33) plants (Figure 9B). Additionally, after five days of inoculation with 5 mL (1 × 10^5^ cfu/mL) spores of *P. capsici*, the transgenic lines (line 4, line 7, and line 8) showed obvious wilting, while the wild-type plants and empty vector transgenic lines (P31 and P33) showed mild or no wilting symptoms (Figure 9C). In addition, the root-stem junction of the transgenic lines (line 4, line 7 and line 8) exhibited constriction (Figure 9C). Previous studies showed that most of the SBP-box genes have a major role in plant growth and development. Therefore, in order to know whether the disease resistance of transgenic lines changed along with seedling age, 65-day-old *N. benthamiana* seedlings were used to identify disease resistance. After two days post-inoculation of *P. capsici*, the detached leaves of wild-type and empty vector (P31 and P33) plants showed a small hygrophanous lesion area, while the hygrophanous lesion area occupied almost half of the leaves in transgenic lines (line 4, line 7 and line 8) with the accumulation of hydrogen peroxide (H_2_O_2_) (Supplementary Figure S2). Hydrogen peroxide (H_2_O_2_) was detected though DAB staining. These results indicated that *CaSBP12* might play a negative regulatory role in plant defense response against *P. capsici* and will not change with seedling age.

#### 2.3.2. Determination of Biochemical Indexes

**Figure 10:**
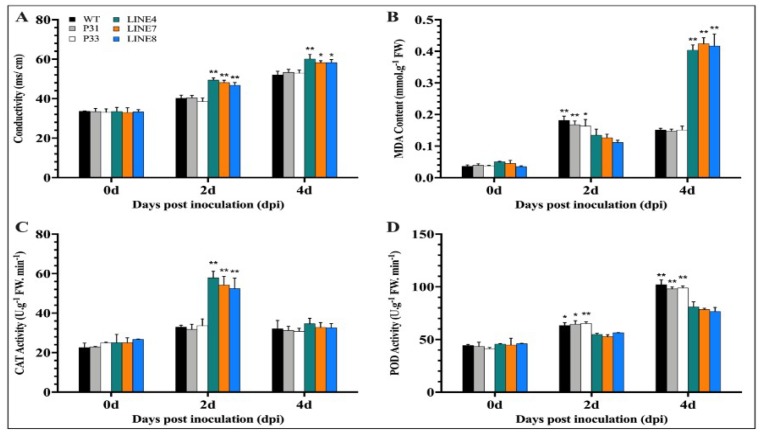
Determination of biochemical indexes of transgenic (line 4, line 7, and line 8), empty vector (P31, P33), and wild-type lines after inoculation with *P. capsici.* (**A**) Conductivity of the transgenic, empty vector, and wild-type lines. (**B**) MDA content of the transgenic, empty vector, and wild-type lines. (**C**) Catalase (CAT) activity of the transgenic, empty vector, and wild-type lines. (**D**) peroxidase (POD) activity of the transgenic, empty vector, and wild-type lines. Data was analyzed between the transgenic lines (line 4, line 7, and line 8) and the wild-type and empty vector transgenic lines (P31 and P33) at the same time point using the least significant difference (LSD). ** or * in the figure indicates that transgenic lines (line 4, line 7, and line 8) had a significant difference (at *p* ≤ 0.01 or *p* ≤ 0.05) compared with control lines (wild-type and empty vector transgenic lines P31 and P33) at the same time point. Mean values and SEs for three replicates are shown.

**Figure 11:**
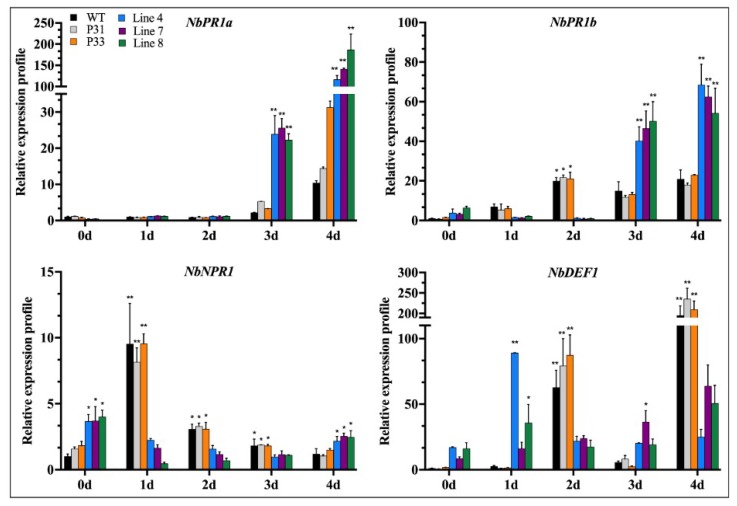
The expression of defense-related genes after inoculation with *P. capsici* in transgenic (line 4, line 7, and line 8), empty vector (P31 and P33), and wild-type lines. The expression levels were analyzed between the transgenic lines (line 4, line 7, and line 8) and the wild-type and empty vector transgenic lines (P31 and P33) at the same time point using the least significant difference (LSD). * represents significant difference at *p* ≤ 0.05, and ** represents highly significant differences at *p* ≤ 0.01. Mean values and SEs for three replicates are shown.

It has been reported that the content of conductivity and MDA in plant can indirectly reflect plant stress resistance during biotic and abiotic stress [19,21]. Catalase (CAT) helps to maintain reactive oxygen homeostasis during biotic and abiotic stress [22]. Peroxidases are known to be activated in response to pathogen attacks, and various roles have been attributed to them, especially roles related to resistance [23]. Therefore, we measured these biochemical indexes after inoculation with *P. capsici*. The conductivity and MDA content of wild-type plants and transgenic lines increased. Besides, the conductivity in the transgenic lines (line 4, line 7, and line 8) was higher than that in the wild-type plants and empty vector transgenic lines (P31 and P33) (Figure 10A). The MDA content in the transgenic lines (line 4, line 7, and line 8) was higher than that in the wild-type plants and empty vector transgenic lines (P31 and P33) on 4 dpi (Figure 10B). The activity of catalase in the transgenic lines was higher than in the wild-type plants and empty vector transgenic lines (P31 and P33) at day two (Figure 10C). In addition, the peroxidase (POD) activity in the transgenic lines (line 4, line 7, and line 8) was lower than that in the wild-type plants and empty vector transgenic lines (P31 and P33) (Figure 10D). In addition, the expression of defense-related genes i.e. *NbDEF* (defensin), *NbNPR1* (non-expressor pathogenesis-related), *NbPR1a* (pathogenesis-related), *NbPR1b* (pathogenesis-related) was also measured. After inoculation with *P. capsici*, the expression of defense-related genes (*NbDEF*: X99403, *NbNPR1*: AF480488, *NbPR1a*: JN247448.1, *NbPR1b*: XM_016587501.1) increased to different degrees (Figure 11). It is notable that the expression of *NbPR1a* and *NbPR1b* in transgenic plants (line 4, line 7, and line 8) was higher than that of wild-type plants and empty vector transgenic lines (P31 and P33), especially at days three and four. However, the expression of *NbNPR1* in transgenic plants (line 4, line 7, and line 8) was decreased during the detection period (Figure 11). The expression of the *NbDEF* was induced in wild-type lines at days two and four, while there was no obvious change in transgenic lines (line 4, line 7, and line 8) except day one and four. These results indicated that the *CaSBP12* plays a negative regulatory role in plant defense response against *P. capsici*.

#### 2.3.3. Disease Index Percent Statistics

**Figure 12:**
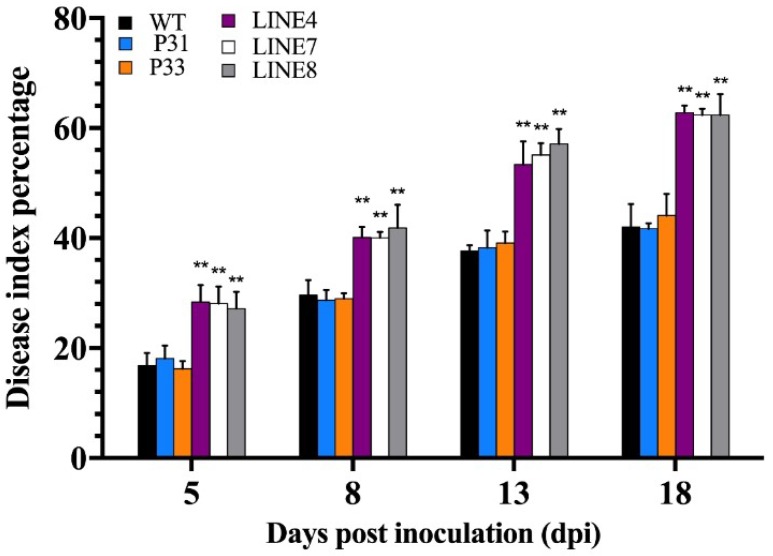
Disease index percent of transgenic (line 4, line 7, and line 8), empty vector (P31, P33), and wild-type lines after inoculation with *P. capsici* and the disease index percent were calculated at 5, 8, 13, and 18 d. The data of disease index percent was analyzed between the transgenic lines (line 4, line 7, and line 8) and the wild-type and empty vector transgenic lines (P31 and P33) at the same time point using the least significant difference (LSD). ** represents highly significant difference at *p* ≤ 0.01. Mean values and SEs are shown.

After inoculation of *P. capsici*, the disease index percent was counted at day five, eight, thirteen, and eighteen. The disease index percent of wild-type, empty vector (P31, P33) and transgenic lines (line 4, line 7, and line 8) increased periodically (Figure 12). Besides, the disease index percent of the wild-type plants and empty vector transgenic lines (P31 and P33) were obviously lower than the transgenic lines (line 4, line 7, and line 8). The detail data of disease index percent is available in Supplementary Table S1. These results indicating that *CaSBP12* transgenic lines (line 4, line 7, and line 8) are more susceptible to *P. capsici* infection compared to wild-type plants.

## 3. Discussion

The SBP-box family gene is a type of specific TF in plants, and it plays important roles in plant growth, signal transduction, and stress response. Previously, we showed that most *CaSBP* genes are induced by *P. capsici* and hormones, but there is no direct evidence proving that they are involved in the resistance to *P. capsici* in pepper [18]. Here, we show that one of the SBP-box genes (*CaSB*P12) plays a negative role in plant defense response against *P. capsici*.

The *CaSBP12* amino acid sequence contains all the features of typical SBP-box proteins including two zinc finger-like structures (C3H, C2HC) and a putative nuclear localization signal [18]. We further confirmed that the *CaSBP12* gene was located in the nucleus (Figure 1). Silencing of *CaSBP12* enhanced the resistance against *P. capsici* infection. With the silencing of *CaSBP12* gene the relative expression of *CaSBP04* was up-regulated because it may be due to close interaction with each other. In addition, we previously identified that both these genes belong to the same gene family (*CaSBP*) indicating their linkage and both are segmentally duplicated [18]. It has also been reported that switchgrass SBP-box transcription factors *PvSPL1* and *PvSPL2* function redundantly to initiate side tillers and affect biomass yield of energy crop [24]. After inoculation with *P. capsici*, the root activity of the control plants decreased and the MDA content increased more than that in the silenced plants (Figure 6D and Figure 7). The lesion area of detached leaves and the diseased index of the silenced plants were smaller than that of control plants (Figure 3 and Figure 8B). It is worth mentioning that after inoculation with the HX-9 strain of *P. capsici*, the disease develops faster than in plants inoculated with the PC strain of *P. capsici* (Figure 3 and Figure 6). Plant immune systems mainly include pathogen-associated molecular pattern (PAMP)-triggered immunity (PTI) and effector-triggered immunity (ETI) [25]. During the compatible interaction of pepper plants with *Xanthomonas campestris pv*. *vesicatoria*, the bacterial effectors cannot be recognized by resistant proteins, then the PTI will be activated and the expression of PR proteins, subsequently establishing of basal defense through SA media signaling pathways [26]. When the expression of these PR proteins is not strong enough to exceed the threshold of the HR in the compatible interaction, the plants will become diseased. During the incompatible interaction of pepper plants with *Xanthomonas campestris pv*. *vesicatoria*, the bacterial effectors will be recognized by resistant proteins and the ETI will be induced [26]. The effector-triggered the immunity, accelerates and amplified PTI response, resulting in disease resistance [25]. However, natural selection drives pathogens to avoid ETI either by shedding or diversifying the recognized effector gene or by acquiring additional effectors that suppress ETI [25]. In compatible plant–microbe interactions, susceptible cell death occurs relatively late during the course of infection [25], providing an explanation for the faster disease development when inoculated with compatible *P. capsici* (HX-9) compared with plants inoculated with incompatible *P. capsici* (PC), and ultimately, pepper plants become diseased. It has been reported that *CaSAR8.2* is a molecular marker for the detection of various pathogenic infections relate to the SA mediated signal transduction pathway [27]. *CaBPR1* and *CaPO1* are related to hypersensitive response [28,29]. Bacterial pathogen infection, abiotic elicitors, and some environmental stressors may play a significant role in the MeJA mediate signal transduction pathway for *CaDEF1* gene expression [30]. Therefore, we chose these genes to research whether the *CaSBP12* gene is related to these signal transduction pathways. After inoculation with *P. capsici*, these PR genes were induced to different degrees. However, the expression of four defense-related genes (*CaPO1*, *CaDEF1*, *CaBPR1*, and *CaSAR8.2*) in control plants peaked early or at the same time as silenced plants and was significantly higher than that of the *CaSBP12*-silenced plants after inoculation with the HX-9 strain of *P. capsici* (Figure 4). Besides, after being inoculated with the PC strain of *P. capsici*, the expression levels of defense related genes in silenced plants was significantly higher than the control plants (Figure 5). It is notable that after inoculation with incompatible *P. capsici* (PC), the expression of PR genes, such as *CaPO1*, *CaBPR1*, and *CaDEF1*, is induced more than in plants inoculated with compatible *P. capsici* (HX-9). It has been reported that PR genes, such as *CaPR10*, *CaBPR1*, and *CaPOA1* are differentially regulated by the SA media signaling pathway, of which the signaling intensity is stronger for ETI than PTI [26]. The expression of defense-related genes in pepper plants is positively related to the resistance of the plants [31]. These indicate that *CaSBP12* genes may be involved in plant defense response against *P. capsici* via the resistance pathway mediated by SA.

Overexpression of *CaSBP12* in *N. benthamiana* enhanced susceptibility to *P. capsici* infection. After inoculation with *P. capsici*, the transgenic lines (line 8, line 7, and line 4) became more susceptible to disease compared to the wild-type and empty vector transgenic lines (P31 and P33) (Figure 9). The MDA and conductivity content and disease index were higher in the transgenic lines (line 8, line 7, and line 4) (Figure 9A,B, and Figure 12). Most of the plant defense enzymes, such as CAT and POD, are closely related to plant resistance. Therefore, in this study, we measured the CAT and POD activity of *P. capsici* post-inoculation. The POD activity in the transgenic lines and wild-type plants increased after inoculation with *P. capsici* while the CAT activity increased at first and then decreased. The CAT activity in the transgenic lines (line 4, line 7, and line 8) was higher than that in the wild-type plants and empty vector transgenic lines (P31 and P33), while the POD activity was lower than that in the wild-type plants and empty vector transgenic lines (P31 and P33) (Figure 10C,D). Study on bacterial blight of rice showed that POD activity was positively correlated with resistance, and the activity of POD in cucumber varieties resistant to downy mildew was higher than in susceptible varieties while the CAT activity was lower compared with susceptible varieties [32,33]. The POD activity in resistant varieties of pepper was also higher than in susceptible varieties [31]. Catalase is not a stable enzyme, and it is inhibited under the high concentrations of H_2_O_2_ produced in plants under stress [23,34]. Therefore, the CAT activity decreased in the later stage of infection after inoculation with *P. capsici*. These findings indicated that *CaSBP12* plays a negative regulatory role in plant defense response against *P. capsici*. It is well-known for transcription factors to play a negative regulatory role in pathogen infection. For instance, overexpression of two WRKY transcription factor family genes (*OsWRKY62.1* and *OsWRKY76.1*) can reduce the resistance to blast fungus *Magnaporthe oryzae* and the leaf blight bacterium *Xanthomonas oryzae* pv. *oryzae* infection in rice [35]. Overexpression of *JcNAC1* in *Jatropha curcas* can improve the sensitivity of plants to pathogen infection and change the expression of some defense-related genes [36]. Over expression of *ATAF2* in *Arabidopsis* can enhance the sensitivity of soil borne disease infection *Fusarium oxysporum* and inhibit the expression of some defense related genes [37]. In *N. benthamiana*, the nucleotide binding rich leucine repeat (N TIR-NB-LRR) can regulate the expression of defense related genes through *SPL6* [38]. The defense-related genes (*NbDEF*, *NbNPR1*, *NbPR1a,* and *NbPR1b*) in *CaSBP12* over expressed plants were differentially expressed, and the expression of *NbPR1a* and *NbPR1b* in transgenic plants (line 4, line 7 and line 8) was higher than that of wild-type plants and empty vector transgenic lines (P31 and P33) (Figure 11). The *NbPR1a* and *NbPR1b* genes were involved in the SA-induced systemic acquired resistance pathway [39], and the *CaSBP12* gene can be inhibited by the SA synthesis inhibitor (paclobutrazol, PBZ) and induced by SA [18]. *P. capsici* is hemi-biotrophic pathogen in nature, which initially follows the SA pathway to cause disease [40]. Later on, it becomes necrotrophic and follows the JA pathway [41]. The SBP gene family could follow the SA pathway [18] antagonistically act and show resistance in the case of knockdown when the genes show resistance. In addition, *VpSBP5* is able to induce resistance to powdery mildew in grapes via the salicylic acid and methyl jasmonate pathways [11]. The *AtSPL2* gene also has the ability to respond to biotic stress through the signaling pathways mediated by salicylic acid and methyl jasmonate [12]. 

## 4. Materials and Methods

### 4.1. Plant Material and Pathogen Preparation

In this study, pepper cultivar AA3 (resistant to the PC strain of *P. capsici* and susceptible to the HX-9 strain of *P. capsici*) was used. Plants were planted in pots with substrate (matrix, vermiculite and perlite mixed with 3:1:1) and grown in a growth chamber at 25 °C (daylight for 16 h)/ 18°C (night for 8 h), humidity 80%. The virulent (HX-9) and avirulent (PC) strains of *P. capsici*, provided by the Capsicum Research Group, College of Horticulture, Northwest A&F University, Xianyang, China, were used in this research. Both of the strains were prepared following the method described by Zhang et al. (2013) [16]. Briefly, both of the strains were first cultured on a petri dish with potato-dextrose agar in the dark at 28 °C for five days. After that, they were incubated under continuous light for seven days. Then the pathogens were overspread on the culture dish and produced a large number of sporangium. Zoospore release was induced by chilling cultures at 4 °C for 30 min and then incubated at room temperature for 1 hour. Zoospore concentration was counted and adjusted to 1 × 10^5^ cfu/mL using the method described by Jin et al. (2016) [1]. Then, 5 mL of this zoospore was used to inoculate the *CaSBP12*-silenced and control pepper plants using the root-drench method as described by Wang (2013) [20].

### 4.2. Subcellular Localization of *CaSBP12*

The ORF of the *CaSBP12* without a termination codon was cloned into a 35S::GFP vector with *Xba*I and *Kpn*I restriction sites to yield the final plasmid 35S::*CaSBP12*::GFP. The CDS of *CaSBP12* was amplified by PCR with *Xba*I and *Kpn*I linker primers *CaSBP12*-GFP2-F and *CaSBP12*-GFP2-R (Supplementary Table S2). The recombinant fusion 35S:*CaSBP12*::GFP and 35S::GFP (used as the control) plasmids were introduced into onion epidermal cells by particle bombardment [42]. After transformation, tissues were incubated on MS agar medium under dark conditions at 28 °C for 24 h and then observed under a fluorescent confocal microscope (Olympus, Tokyo, Japan).

### 4.3. Production of CaSBP12-silenced Pepper Plants by Virus-Induced Gene Silencing (VIGS)

The TRV-based VIGS system was used for the silencing of *CaSBP12*, as previously described [19]. To generate the pTRV2:*CaSBP12* construct, a 250-bp fragment of the *CaSBP12* was amplified using the specific primer, and its specificities were assessed using NCBI Primer BLAST (Supplementary Table S2). The obtained product was sequenced by Sangon-Biotech Company (Shanghai, China) and then cloned into a TRV2 vector using the double digested method with *Bam*HI and *Kpn*I enzymes. The recombined vector pTRV2:*CaSBP12*, pTRV2 (negative control), pTRV2:*CaPDS* (phytoenedesaturase, positive control), and pTRV1 were transformed into *Agrobacterium tumefaciens* strain GV3101 using the freeze-thaw method. Pepper seedlings at the two true leaves (40 days after sowing) stage were used for the silencing of *CaSBP12* as described by Zhang et al. (2013) [16]. All the injected plants were grown in a growth chamber with the having same growing conditions as described by Wang (2013) [20]. Forty-five days post-infiltration, leaf and root samples from the control and silenced plants were collected to measure the silencing efficiency. After that, the assay of the detached leaves was conducted as described by Zhang (2015) [43]. That is, the detached leaves of *CaSBP12*-silenced and control plants were placed in petri-dishes respectively. Filter paper with sterile water underneath the leaves was used to maintain appropriate humidity in the dish. Then, the plug (8 mm diameter) of *P. capsici* was used to inoculation the detached leaves. After that, the detached leaves were placed in the incubator at 25 °C (daylight for 16 h)/ 18 °C (night for 8 h), humidity 80%. During this period, the diseased symptoms of detached leaves was recorded and photographed. Five milliliters of 1 × 10^5^ cfu/mL zoospore of the virulent (HX-9) and avirulent (PC) strains of *P. capsici* were used to inoculate the silenced and control plants, respectively, using the root-drench method [20]. Briefly, at a distance of about 3 cm from the root of the seedling, a glass rod (0.8 cm diameter) was used to pierced a 3cm deep hole and then 5 mL of 1×10^5^ cfu/mL zoospore was injected into the hole. After that, we randomly selected fifteen members of the *CaSBP12*-silenced plants^,^ roots from the *CaSBP12*-silenced plants and control plants, respectively, and randomly divided them into three parts and stored at −80 °C for the detection of defense related genes. For the roots collected, we moved the plants together with the substrate from the pot to our prepared distilled water (distilled water was prepared three days before sampling and placed in the same condition with the *CaSBP12*-silenced and control plants) and gently cleaned the substrate, and then gently dried it with moisture-absorbent paper.

### 4.4. Nicotiana Benthamiana Transformation

All the encoding regions of *CaSBP12* was cloned into the PBI121-GUS vector with *Xba*I and *Bam*HI restriction enzyme sites to yield the final plasmid PBI121-*CaSBP12*-GUS for genetic transformation (the primer used for this experiment is given in Supplementary Table S2). The *Agrobacterium*-mediated tobacco leaf disc transformation method was used to obtain the overexpressing *CaSBP12* transgenic *N. benthamiana* plants [44]. Eight kanamycin-resistant lines of transgenic *N. benthamiana* plants harboring the PBI121-*CaSBP12*-GUS construct or the PBI121-GUS construct were selected and confirmed using real time polymerase chain reaction (RT-PCR) during T2 generation. Seeds of T1 plants were collected from regenerated T0 plants, and seedlings of T2 lines were further selected on MS agar plates containing 100 μg/mL kanamycin. T3 plants were used for further analyses.

### 4.5. RNA Extraction and Quantitative Real-Time Polymerase Chain Reaction (qRT-PCR)

RNA was extracted according to the method described by Guo et al (2012) [45]. cDNA was synthesized using Prime Script Kit (Takara, Dalian, China) following the manufacturer’s instructions. The cDNA concentration used for qRT-PCR was 50 ng/µL. The iCycleriQ™ Multicolor PCR Detection System (Bio-Rad, Hercules, CA, USA) machine was used for qRT-PCR and the procedure was designed following Zhang et al. [18]. Briefly, the qRT-PCR cycling conditions were as follows: pre-denaturation at 95 ℃ for 1 min, followed by 40 cycles of denaturation at 95 ℃ for 10 s, annealing at 56 °C for 30 s, and extension at 72 °C for 30 s. The fluorescent signal was measured at the end of each cycle, and melting curve analysis was performed by heating the PCR product from 56 to 95 °C in order to verify the specificities of the primers. The actin mRNA (*CaActin2*, accession No. AY572427) from pepper and the actin-97 mRNA (*Nbactin-97*, accession No. LOC109206422) from *N. benthamiana* were used as references, respectively [46,47]. All the primer specificities used for the qRT-PCR were assessed using NCBI Primer BLAST (Supplementary Table S3). Three independent biological replicates were carried out. The relative expression of genes was calculated using the 2^−^^△△*C*t^ method described by Schmittgen and Livak (2008) [48].

### 4.6. Determination of Root Activity

After inoculation of the compatible (HX-9) and incompatible (PC) strains of *P. capsici*, root activity of the silenced and control plants was measured at different time points using the triphenyl-tetrazolium chloride (TTC) method as described by Zhang (2013) [31]. Root tips (0.3–0.5 g) post-inoculation with *P. capsici* were collected from the silenced and control plants, rinsed with water and gently dried with moisture absorbent paper. Then, the root tissues were incubated in 20 mL 1:1 (*v*/*v*) mixture of 1% TTC solution and 0.l M phosphate buffer (pH 7.0) at 37 °C for 1h in the dark. After incubation, 2 mL sulfuricacid (1 M) was added to stop the reaction. The TTF (a derivative of TTC from the reduced reaction) was extracted using the method described by Jin et al. (2016) [1]. Briefly, the root samples were rinsed twice with distilled water and ground in mortar with 3 mL ethyl acetate, in which the extracts of TTF (a derivative of TTC from the reduced reaction) were obtained and transferred to a 10 mL volumetric flask. The residues were rinsed with ethyl acetate and mixed with the earlier extracts, and then the final volume was adjusted to 10 mL. The absorption of the extraction was measured with a spectrophotometer at 485 nm. The reduction amount of TTC was obtained though the standard curve. Then, the root activity was calculated using the formula described by Jin et al. (2016) [1]. Three biological replicates and three technical replicates were used in this experiment.

### 4.7. Determination of Malondialdehyde (MDA) Content

The MDA content of *P. capsici* post inoculation was measured using the modified method of colorimetric determination with thiobarbituric acid [19]. Leaves of the *CaSBP12*-silenced and control plants of pepper were used for MDA determination. Besides, leaves of transgenic *N. benthamiana* plants and wild-type plants were also used for MDA determination. The crude enzyme was extracted using 0.2 M phosphate buffer (pH 7.8). Reaction system is 2 mL, crude enzyme + 5 mL, 0.5% Thiobarbituric acid (TBA), and then boiled for 10 min. After cooling, samples were centrifuged for 10 min (5000 rpm), and then the absorbance was measured at 600, 532 and 450 nm.

### 4.8. Determination of Ion Conductivity and Histochemical Staining

After *P. capsici* inoculation, the ion conductivity was measured using the method described by Cai et al. (2015) [21,49,50]. Briefly, leaf discs (0.5 cm diameter) were excised from transgenic and wild-type lines after inoculation with *P. capsici* at day zero, one, two, three, and four, using a cork borer and subsequently floated on 20 mL of sterile double-distilled water and incubated for 1 h at room temperature. Electrolyte leakage values were determined by DDS-307 conductivity meter (Leizi, Shanghai, China). After inoculation with *P. capsici*, the detached leaves were stained with 3, 3′-diaminobenzidine (DAB) for H_2_O_2_ detection [51]. For DAB staining, the leaves were stained overnight in 1 mg mL^−1^ of DAB. Then the chlorophyll was removed for proper visualization of the stain. This was done by immersing the leaves in absolute ethanol and heating in a boiling water-bath for 30 min.

### 4.9. Determination of Antioxidant Activity

After inoculation with the HX-9 strain of *P. capsici*, leaves of transgenic *N. benthamiana* plants and wild-type plants were sampled at day zero, one, two, three and four forcrude extraction. The medium of crude extraction for the determination of peroxidase (POD) and catalase (CAT) activity were 0.2 M phosphate buffer (pH 7.8) and 0.05 M Tris-HCl buffer (pH7.0), respectively. Peroxidase activity was measured using the guaiacol method (Reaction system: 0.1 mL crude enzyme + 2 mL, 0.3% H_2_O_2_ + 0.9 mL, 0.2% guaiacol) [52]. Catalase activity was measured as described by Aebi (1984) [53]. The reaction system of CAT was as follows: 1.0 mL tris-HCl + 1.7 mL distilled water + 0.1 mL, 100 µM H_2_O_2_ + 0.5 mL crude enzyme.

### 4.10. The Disease Index Percent Statistics

The disease index percent was calculated following Zhang (2009) [54]. After inoculation with the HX-9 strain of *P. capsici*, the plant disease was categorized into 4 levels. They are as follows: Level 0: no symptoms; Level 1: the bottom leaves occasionally fall off or significant plant defoliation occurs; Level 2: blackening of stem base and all leaves falloff except at growing points; Level 3: death of the whole plant. The disease in the transgenic lines was divided into 5 levels after inoculation with the HX-9 strain of *P. capsici* as follows: Level 0: no symptoms; Level 1: wilting of the lower leaves; Level 2: wilting of the upper plant leaves, too; Level 3: constriction of the residual part of the stem or lodging due to stem base constriction; Level 4: whole plant death. The disease index percent was calculated using the following formula: Disease index percent = ((∑the numerical grade of disease × number of disease plants of this grade)/(the highest grade of disease × total number of surveys)) ×100.

### 4.11. Statistical Analysis

Statistical analysis was performed using the data processing system, a software package, which has the complete function of experimental design and statistical analysis (DPS7.05, Hangzhou, China). The means were analyzed using the least significant difference (LSD) value. *p* ≤ 0.05 or *p* ≤ 0.01 was considered significantly different. All experiments were performed and analyzed separately with at least three biological replicates.

## 5. Conclusions

Silencing and over-expression of *CaSBP12* gene induced the transcript level of defense-related genes. Furthermore, the silencing effect of *CaSBP12* in pepper can enhance the resistance against *P. capsici*, as well as its over-expression in the *N. benthamiana* increase its susceptibility. So, this implies that the *CaSBP12* gene plays a negative role in plant defense response against *P. capsici*. We believe that our work provides evidence for further studies on the SBP-box gene on pathogen infection.

## Supplementary Materials

Supplementary materials can be found at http://www.mdpi.com/1422-0067/20/1/48/s1.

## Abbreviations

SBP-box	Squamosa-promoter binding protein
MDA	Malondialdehyde
*CaPO1*	Pepper peroxidase-like gene
*CaBPR1*	Pepper pathogenesis-related (PR)-1 protein
*CaDEF1*	Pepper defensin gene
EC	Electrical conductivity
*NbDEF*	Nicotiana benthamiana defensin gene
*NbNPR1*	*Nicotiana benthamiana* non-expressor of PR genes
*NbPR1a*	*Nicotiana benthamiana* pathogenesis-related protein PR1a
*NbPR1b*	*Nicotiana benthamiana* pathogenesis-related protein PR1b
