# Supplementary material for: A Novel Transcription Factor CaSBP12 Gene Negatively Regulates the Defense Response against Phytophthora capsici in Pepper (Capsicum annuum L.)
Source: Int J Mol Sci. 2018 Dec 22;20(1):48. doi: 10.3390/ijms20010048 (PMC6337521; doi:10.3390/ijms20010048)
Supplement: Supplementary file 1 [file ijms-20-00048-s001.pdf]

SUPPLEMENTARY FIGURES AND TABLES

Supplementary Figure S1. The average diameter of leaf lesion (cm) of transgenic (line 4, line 7, and line 8), empty vector (P31 and P33), and wild-type (WT) lines after inoculated with *P. capsici* 44 hours. Bars with different lower-case letters indicate significant differences using the least significant difference (LSD) value ($P \leq 0.05$). Mean values and SEs are shown.

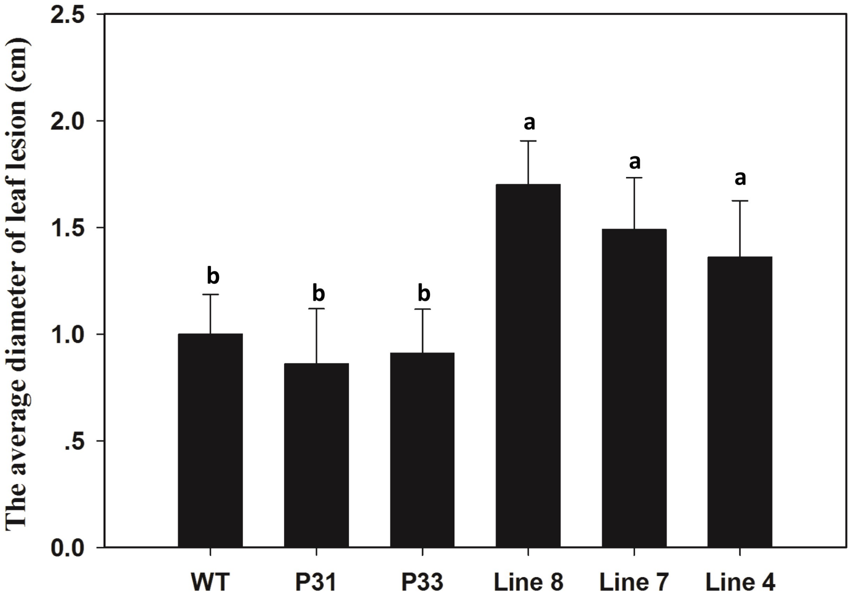

Supplementary Figure S2. Phenotypes of detached leaves of transgenic (line 4, line 7, and line 8), empty vector (P31 and P33), and wild-type (WT) lines and DAB staining *P. capsici* post-inoculation. (A) Phenotypes of detached leaves of transgenic, empty vector, and wild-type lines after inoculation with *P. capsici*. (B) Phenotypes of the detached leaves stained with DAB after inoculation with *P. capsici*. *N. benthamiana* plants at the age of 65 days were used for this experiment. The diameter of the plug of *P.capsici* used in this experiment is 0.4 cm.

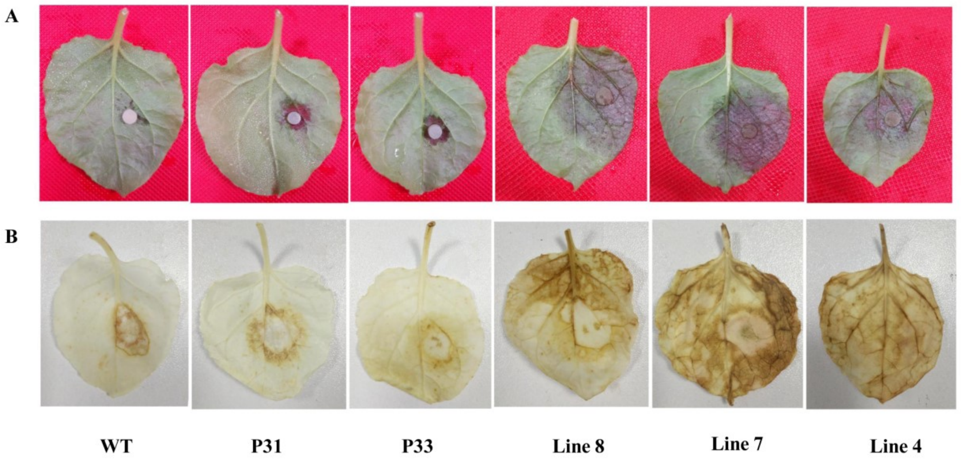

Supplementary Table S1. The detail data of disease index percent of transgenic (line 4, line 7, and line 8), empty vector (P31 and P33), and wild-type (WT) lines after inoculated with *P. capsici*.

	Days of treatment	0 level plants	1 level plants	2 level plants	3 level plants	4 level plants	Total number of treatment plants	Disease index percent
WT	5	9	7	2	1	0	19	18.42
	8	7	6	0	5	0		31.58
	13	7	5	1	3	3		36.84
	18	7	5	0	1	6		42.11
P31	5	12	2	0	3	0	15	18.33
	8	11	0	1	0	4		30.00
	13	7	2	1	1	4		38.33
	18	7	2	0	1	5		41.67
P33	5	11	2	0	3	0	16	17.19
	8	8	3	1	2	2		29.68
	13	6	3	2	2	3		39.06
	18	5	2	3	2	4		46.88
Line 4	5	11	11	2	8	0	32	30.47
	8	10	9	1	6	6		41.41
	13	4	8	5	8	7		53.13
	18	4	5	7	4	12		61.72
Line 7	5	7	7	2	3	0	19	30.26
	8	5	6	1	5	2		40.79
	13	2	5	4	2	6		56.58
	18	1	5	3	3	7		63.16
Line 8	5	10	10	3	6	0	29	29.31
	8	9	3	4	11	2		44.83
	13	5	6	2	10	6		55.17
	18	4	6	3	3	13		62.93

WT	5	19	1	8	0	0	28	15.18
	8	17	1	3	4	3		27.69
	13	16	1	0	2	9		38.39
	18	15	1	0	2	10		41.96
P31	5	10	8	2	1	0	21	17.86
	8	9	6	3	1	2		27.38
	13	7	6	2	2	4		38.10
	18	7	5	1	4	4		41.67
P33	5	12	9	1	1	0	23	15.21
	8	10	7	1	2	3		28.26
	13	8	6	2	2	5		39.13
	18	8	5	2	3	5		41.30
Line 4	5	8	8	3	3	0	22	26.14
	8	7	5	4	3	3		38.63
	13	5	1	7	4	5		53.41
	18	4	1	5	3	9		63.64
Line 7	5	14	2	9	3	0	28	25.89
	8	11	2	6	6	3		39.29
	13	9	1	3	7	8		53.57
	18	7	1	2	8	10		61.61

Level 0: no symptoms; Level 1: wilting of the lower leaves; Level 2: wilting of the upper plant leaves too; Level 3: constriction of the residual part of the stem or lodging due to stem base constriction; Level 4: whole plant death.

Supplementary Table S2. Primers names and their sequences used for vector construction in this study.

Oligo Name	Primer Abbreviation	Primer Sequence (5'-3')
	CaSBP12-gfp2-F	GCTCTAGAATGTTGGACTATGACTGGGGAG
	CaSBP12-gfp2-R	GGGGTACCTGGTCTTTGCCTAAAACAATCC
CaSBP12	CaSBP12-vigs-F	CGGGATCCATCCTCCGTTATGCTTTCTGGC
	CaSBP12-vigs-R	GGGGTACCTACCTTGGGAATGGGTGAAACA
	CaSBP12-PBI121-F	GCTCTAGAATGTTGGACTATGACTGGGGAG
	CaSBP12-PBI121-R	CGGGATCCTGGTCTTTGCCTAAAACAATCC

Supplementary Table S3. Primers names and their sequences used in this study for quantitative real-time PCR.

Oligo Name	Primer Abbreviation	Primer Sequence (5'-3')
	RTSBPzunla-12-F	ATCCTCCGTTATGCTTTCTGGC
	RTSBPzunla-12-R	TACCTTGGGAATGGGTGAAACA
CaSBP12	RTCaSBP12-VIGS-F	GTTTCACCCATTCCCAAGGTAATT
	RTCaSBP12-VIGS-R	TAGTACGTCGGTAAAGTCGATTAACAA
Nbactin-97	NbACTIN-F	TATGGAAACATTGTGCTCAGTGG
	NbACTIN-R	CCAGATTCGTCATACTCTGCC
CaActin2	CaActin2-F	TCCACCTCTTCACTCTCTGCTC
	CaActin2-R	TGACCCATCCCTACCATAACAC
CaSBP04	RTSBPzunla-4-F	ATGACAAATTATCAACACCAGCA
	RTSBPzunla-4-R	AAGCAAAATACGTTCTCCCG
CAPO1	CAPO1-F	GGCGCCAGGATTGCTGACAA
	CAPO1-R	GTGGACATAATCCTCGAAGC
CADEF	CADEF-F	CAAGGGAGTATGTGCTAGTGAGAC
	CADEF-R	TGCACAGCACTATCATTGCATAC
CASAR82	CASAR82-F	CAGGGAGATGAATTCTGAGGC
	CASAR82-R	CATATGAACCTCTATGGATTTCTG
CABPR1	CAPR1-F	CAGGATGCAACACTCTGGTGG
	CAPR1-R	ATCAAAGGCCGGTTGGTC
NbDEF	NbDEF-RT-F	AACTTGTGAGTCCCAGAG
	NbDEF-RT-R	GGATACCTTTCTACCACC
NbNPR1	NbNPR1-RT-F	TTACTTCACTGAAACGCCT
	NbNPR1-RT-R	CACTTCCTTTAATTCCACCT
NbPR1a	NbPR1a-RT-F	GTAATATCCCACTCTTGCCG
	NbPR1a-RT-R	ATGAAATCGCCACTTCCCTC
NbPR1b	NbPR1b-RT-F	TCAAGCTCAAAACTCTCCCC
	NbPR1b-RT-R	CCACATCTTTACTGCTCCCG
